# Chikungunya Virus Spreads in the Americas — Caribbean and South America, 2013–2014

**Published:** 2014-06-06

**Authors:** Marc Fischer, J. Erin Staples

**Affiliations:** 1 Arboviral Diseases Branch, National Center for Emerging and Zoonotic Infectious Diseases, CDC

In December 2013, the World Health Organization reported the first local transmission of chikungunya virus in the Western Hemisphere, with autochthonous cases identified in Saint Martin (1). Since then, local transmission has been identified in 17 countries or territories in the Caribbean or South America (Anguilla, Antigua and Barbuda, British Virgin Islands, Dominica, Dominican Republic, French Guiana, Guadeloupe, Guyana, Haiti, Martinique, Puerto Rico, Saint Barthelemy, Saint Kitts and Nevis, Saint Lucia, Saint Martin, Saint Vincent and the Grenadines, and Sint Maarten). As of May 30, 2014, a total of 103,018 suspected and 4,406 laboratory-confirmed chikungunya cases had been reported from these areas.* The number of reported cases nearly doubled during the previous 2 weeks. More than 95% of the cases have been reported from five jurisdictions: Dominican Republic (38,656 cases), Martinique (30,715), Guadeloupe (24,428), Haiti (6,318), and Saint Martin (4,113). The highest incidences have been reported from Saint Martin (115 cases per 1,000 population), Martinique (76 per 1,000), Saint Barthelemy (74 per 1,000), and Guadeloupe (52 per 1,000). Further expansion of these outbreaks and spread to other countries in the region is likely.

Chikungunya virus is a mosquito-borne alphavirus transmitted primarily by *Aedes aegypti* and *Aedes albopictus* mosquitoes (1–3). These vectors also transmit dengue virus and are found throughout much of the Americas, including parts of the United States. Humans are the primary amplifying host for chikungunya virus, and most infected persons develop symptomatic disease (2). The most common clinical findings are acute onset of fever and polyarthralgia. Joint pains are usually bilateral and symmetric; they can be severe and debilitating. Mortality is rare and occurs mostly in older adults.

Chikungunya outbreaks previously have been documented in countries in Africa, Asia, Europe, and the Indian and Pacific Oceans. Before the cases on Saint Martin, the only chikungunya cases identified in the Americas were in travelers to or from known endemic areas. None of these cases resulted in local transmission or outbreaks.

Chikungunya is not a nationally notifiable disease in the United States. However, chikungunya cases can be reported to ArboNET, a national passive surveillance system for arthropod-borne diseases. During 2006–2013, studies identified an average of 28 persons per year (range: 5–65) with positive tests for recent chikungunya virus infection from one of the four U.S. laboratories that perform testing. All were travelers visiting or returning to the United States from affected areas, mostly in Asia (1,4). Only 23% of the cases were reported to ArboNET. Beginning in 2014, cases have been identified in travelers returning from the Caribbean. As of June 2, a total of 28 chikungunya cases had been reported to ArboNET from U.S. states and territories. On May 30, the Puerto Rico Department of Health reported their first locally transmitted case; local transmission has not been identified in other U.S. states or territories. The remaining U.S. cases have occurred in travelers returning from affected areas, including 26 travelers returning from the Caribbean (Dominica, Dominican Republic, Haiti, Martinique, Saint Martin, and Sint Maarten) and one traveler returning from Asia (Indonesia). With the recent outbreaks in the Caribbean and the Pacific, the number of chikungunya cases among travelers visiting or returning to the United States from affected areas will likely increase. These imported cases could result in local spread of the virus in other parts of the United States.

Chikungunya virus infection should be considered in patients with acute onset of fever and polyarthralgia, especially travelers who recently returned from areas with known virus transmission. Chikungunya virus diagnostic testing currently is performed at CDC, three state health departments (California, Florida, and New York), and one commercial laboratory (Focus Diagnostics).

No specific treatment, vaccine, or preventive drug is available for chikungunya virus infection. Treatment is palliative and can include rest, fluids, and use of analgesics and antipyretics (1,3). Most patients’ symptoms improve within 1 week. In some persons, joint pain can persist for months (2,3). The best way to prevent chikungunya virus infection is to avoid mosquito bites: use air conditioning or screens when indoors, use insect repellents, and wear long sleeves and pants when outdoors. Persons infected with chikungunya virus should be protected from mosquito exposure during the first week of illness to prevent further spread of the virus.

Health-care providers are encouraged to report suspected chikungunya cases to their state or local health department to facilitate diagnostic testing and mitigate the risk for local transmission. CDC and the Council of State and Territorial Epidemiologists urge health departments to perform surveillance for chikungunya cases in returning travelers and be aware of the risk for possible local transmission in areas where *Aedes* species mosquitoes are currently active. State health departments are encouraged to report confirmed chikungunya virus infections to CDC through ArboNET (1).

## Footnotes

* *Suspected case:* patient with acute onset of fever >101°F (>38°C) and severe arthralgia or arthritis not explained by other medical conditions, and who resides or has visited epidemic or endemic areas within 2 weeks before the onset of symptoms. *Confirmed case:* a suspected case with laboratory evidence of recent chikungunya virus infection (i.e., viral isolation, reverse transcription–polymerase chain reaction, immunoglobulin M antibodies, or a fourfold or greater increase in virus-specific neutralizing antibody titers) (3).
